# Evolutionary History of Multiple Dural Fistula

**DOI:** 10.1177/2324709616683722

**Published:** 2016-12-01

**Authors:** Braulio Martinez-Burbano, Edgar Patricio Correa Diaz, Carolina Jácome Sánchez

**Affiliations:** 1 Carlos Andrade Marin Hospital, Quito, Ecuador

**Keywords:** multiple dural fistula, headache, intracranial hypertension, dementia

## Abstract

Intracranial dural arteriovenous fistulas (DAVFs) are abnormal communications between arteries and veins or dural venous sinuses, which sit between the sheets of the dura. They represent 10% to 15% of intracranial vascular malformations. Clinical manifestations and prognosis depend on the pattern of venous drainage and location. The clinical presentation of DAVF may be mistaken for vascular or nonvascular brain pathologies. For that reason, within the differential diagnosis come a wide range of conditions, such as secondary headaches, encephalopathies, dementias including those with rapid progression, neurodegenerative diseases, inflammatory processes, or tumors typically at the orbital level or in the cavernous sinus. Diagnosis requires a high degree of suspicion because of the multiplicity of symptoms and presentations, making this pathology an entity that provides a major challenge for clinicians, yet early and multidisciplinary treatment of high-grade fistulas improve the possibility of avoiding poor or unfavorable outcomes for the patient.

## Introduction

Intracranial dural arteriovenous fistulas (DAVFs) are abnormal communications between dural arteries and veins (meningeal or cortical veins) or venous sinuses, which are located between the sheets of the dura. In adults, the DAVFs are acquired conditions.^1-3^ The pathogenesis of DAVF is not very clear and is generally idiopathic. However, several factors associated with their formation have been identified, including venous sinus thrombosis, cranial trauma, infection, cranial surgery, and hypercoagulability.^4-8^ Recently, a new theory has been proposed that it is an inflammation of the emissary vein at the point of entry into the dura, which induces venous dilation and neovascularization.^8^ DAVFs account for 10% to 15% of intracranial vascular malformations.^1,9^ They have a slight predominance in females, with a ratio of 1.65:1, and the average age of onset is around 60 years.^10,11^

DAVFs may be asymptomatic or present a wide variety of symptoms and signs, which may be ocular, focal neurological deficit, chronic headache, pulsatile tinnitus, seizures, or intracranial hemorrhage.^1,3,10-15^ Additionally, there may be cognitive impairment, affecting the domains of memory, calculation, orientation, visuospatial function, and language, even reaching a state of dementia.^16-18^ Intracranial hypertension is another clinical manifestation and is related to hydrocephalus and passive venous congestion also expressed as a venous hypertensive encephalopathy.^1,10,19^ Cases with unusual clinical manifestations, such as gait ataxia, parkinsonism, and myoclonus have also been reported.^7,20,21^

The clinical presentation varies according to location, pattern, and direction of the flow of venous drainage and can have a benign or aggressive nature, the latter being in relation to cortical cortical venous drainage (CVD).^5,22,23^ One factor that defines a DAVF as high grade is the presence of CVD, which leads to an increased risk of brain hemorrhage and neurological ischemic injury. Therefore, within the group of high-grade fistulas, there is a subset of CVD-symptomatic fistulas, which come with a risk of intracranial hemorrhage in 7.4% to 7.6% of cases.^24^ The most common locations in relation to the venous drainage appear in the following order of frequency: cavernous sinus, transverse sinus-sigmoid, tentorium, anterior cranial fossa, and superior sagittal sinus.^1,11^ However, there are multiple dural arteriovenous fistulas, which represent 2% to 7% of all DAVFs.^6,25^

In this article, we present the case of a patient with multiple dural arteriovenous fistula, which, in its evolution, presented a transformation of a fistula from low to high grade, showing a clinical condition that progressed from headaches to a vegetative state.

## Clinical Case

**Figure 1.**
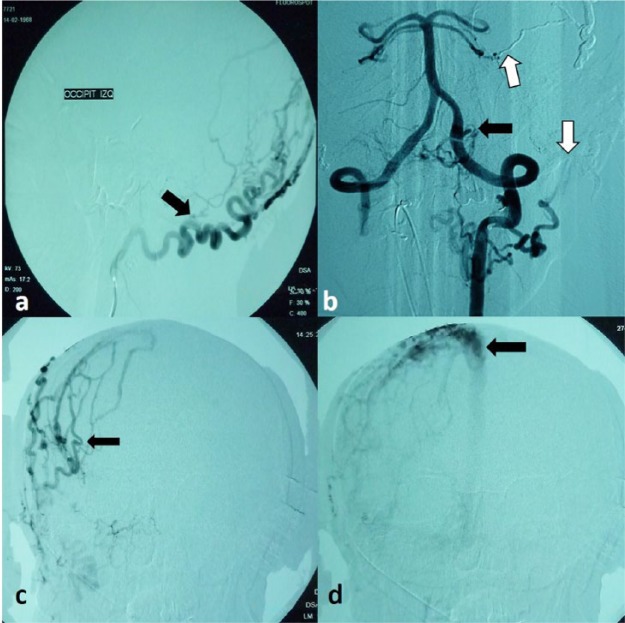
Cerebral angiography. (a) Left occipital artery (black arrow). (b) Branches of the left vertebral artery (black arrows) with embolization material (white arrow). (c) Branches of the superficial temporal artery (black arrows). (d) Drainage to the superior sagittal sinus (black arrows).

Male patient, age 45, with a history of treatment with levothyroxine for multinodular goiter. In 2008, progressively dilated superficial facial veins at the bilateral temporal level were noticed. Three years later (2011), he presented a persistent front pulsatile and left retro-ocular headache of moderate intensity, with greater intensity in the morning and progressively less intensity throughout the day. On suspicion of a dural fistula, an angiography was performed, which showed the presence of a dural fistula of the afferent arterial branches of the external carotid artery. The largest tributary of the dural fistula was dependent on the left occipital artery and to a lesser extent on the internal maxillary artery, superficial temporal artery, and some smaller branches of the ascending pharyngeal artery. Also observed were other branches dependent on the left vertebral artery with drainage to the superior sagittal venous sinus and transverse/sigmoid sinus, so it was diagnosed as multiple arteriovenous dural fistula Cognard Type IIa/Borden Type I (Tables 1 and 2). Cerebral angiography with 2 sessions of embolization was performed, occluding the occipital left artery and branches of the left vertebral artery (Figure 1).

**Table 1.** Borden Classification of Dural Arteriovenous Fistula^2^.

1. Venous sinus drainage anterograde flow
2. Venous sinus drainage retrograde flow into leptomeningeal veins
3. Drainage into leptomeningeal veins

**Table 2.** Cognard Classification of Dural Arteriovenous Fistula^2^.

I. Main sinus drainage, anterograde flow
IIa. Main sinus drainage into a sinus
IIb. Main sinus drainage into leptomeningeal veins
IIa+b. Main sinus drainage into sinus and leptomeningeal veins
III. Direct leptomeningeal venous drainage without venous ectasia^a^
IV. Direct leptomeningeal venous drainage with venous ectasia
V. Spinal venous drainage

a Venous ectasia = venous dilatation >5 mm in diameter or 3 times larger than the diameter of the draining vein.

**Figure 2.**
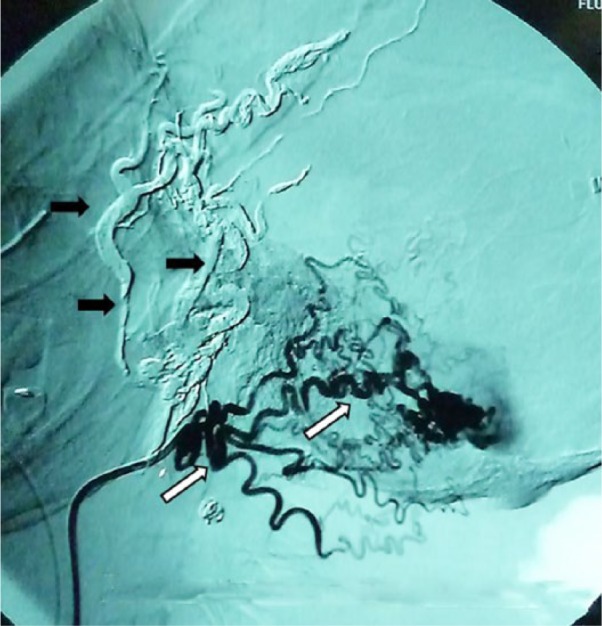
Cerebral angiography. Recanalization of left occipital artery (white arrows). Embolization of branches of occipital artery with onyx (black arrows).

Over the next 3 months, the patient continued to experience increased headache frequency and intensity, which worsened with Valsalva maneuvers. Moreover, bilateral tinnitus more intense on the left side was presented. Physical examination showed the presence of an audible bruit with thrill (systolic) at the bilateral occipital level. A new cerebral angiography was performed, which found a recanalization of the left occipital artery in addition to new arterial feeders and significant dilation of the right occipital, superficial temporal, and middle meningeal arteries. New embolization sessions of the middle meningeal and occipital artery were scheduled; 90% of dural fistula was closed after embolization. The symptoms such as headache and tinnitus reached total improvement (Figure 2).

**Figure 3.**
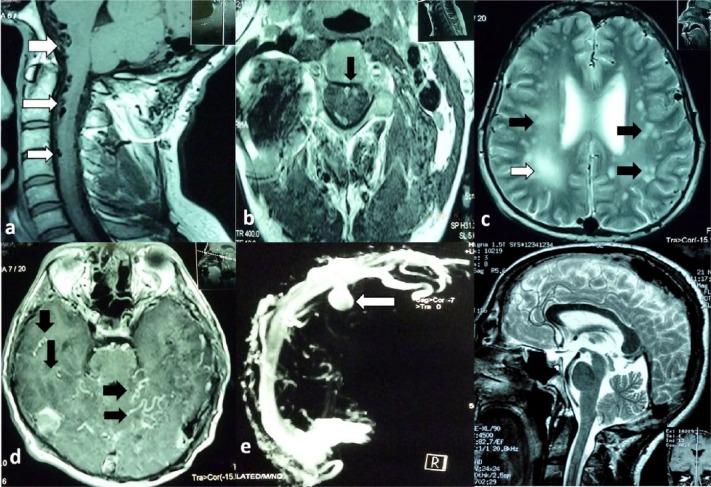
MRI of cervical spinal. (a) Sagittal T1-weighted demonstrate perimedullary venous plexus dilatation (white arrows) and venous pouch at the infratentorial level (head of white arrow). (b) Axial T1-weighted showed perimedullary dilated venous plexus. MRI of brain. (c) Axial T2-weighted reveal multiple foci of leukoaraiosis (black arrows), edema of white matter (white arrows), and venous ischemia (head of white arrow). (d) Axial with Gd showed vascular enhancement (black arrows). (e) MRI venography demonstrate presence of venous pouch at the supratentorial level (white arrows). (f) MRI brain without changes of white matter (before evolution of dural fistula).

In 2012, the patient’s headaches returned and were accompanied by eye pain and progressive loss of bilateral visual acuity, in addition to vomiting and difficulty in walking. On neurologic examination, spasticity of the lower limbs with hyperreflexia and bilateral clonus were found. The indirect ophthalmoscopy revealed bilateral papilledema and normal intraocular pressure. A lumbar puncture showed a cerebrospinal fluid opening pressure of 43 cm H_2_O, which was identified as an intracranial hypertension syndrome, as a result of venous hypertension due to dural fistulas. The study of magnetic resonance imaging (MRI) of the cervical spine showed a slight diffuse pericentral widening of the spinal cord from C2 to C6, evident in T1- and T2-weighted sequences. MRI with gadolinium (Gd), MR angiography, and MR venography identified multiple foci of leukoaraiosis and edema in white matter, especially in the right hemisphere. Moreover, there is vascular enhancement and several venous pouch (venous reservoirs) at the supra and infratentorial levels (Figure 3).

**Figure 4.**
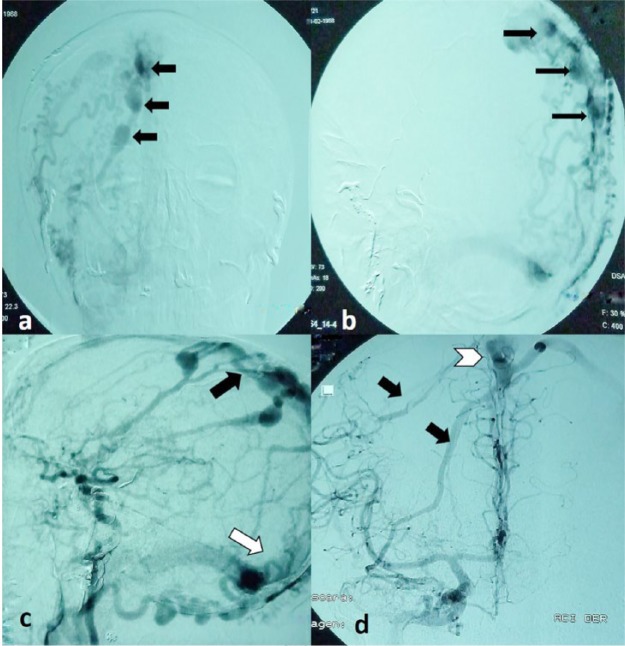
Cerebral angiography. (a and b) Dilated arterial branches of the external carotid artery (black arrows) and transverse sinus (white arrow). (c) Drainage from branches of the external carotid artery to superior sagittal (black arrow) and transverse sinus (white arrow). (d) Arterial feeders of the internal carotid artery (black arrows) with superior sagittal sinus drainage (head of white arrow).

On conducting a new angiographic study, persistence of dural fistula with dilated branches of the external carotid artery was observed, along with new arterial feeders from external, vertebral, carotid, and left ascending cervical arteries draining into the transverse sinus and sigmoid. In addition, arterial feeders of the internal carotid (sylvian) and external carotid to superior sagittal sinus with retrograde flow to cortical veins and significant leptomeningeal venous hypertension was noted. Dilation and hypertension in the perimedullary venous plexus and anterior spinal vein was also found, extending approximately to T3. Diagnosis was of a multiple dural arteriovenous fistula Cognard type V/Borden Type III, which was subjected to a new endovascular embolization treatment (Figure 4).

A month later, the patient experienced mood changes, including permanent irritability, along with cognitive impairment of memory and calculation. On neuropsychological evaluation, a cortical-subcortical cognitive impairment was found, with a prevalence in the subcortical region. Eventually, the patient’s memory worsened and there were problems related to attention, overall disorientation, and constructional apraxia, along with a marked slowing in cognitive processing speed, visuospatial defects, and failure of executive function.

**Figure 5.**
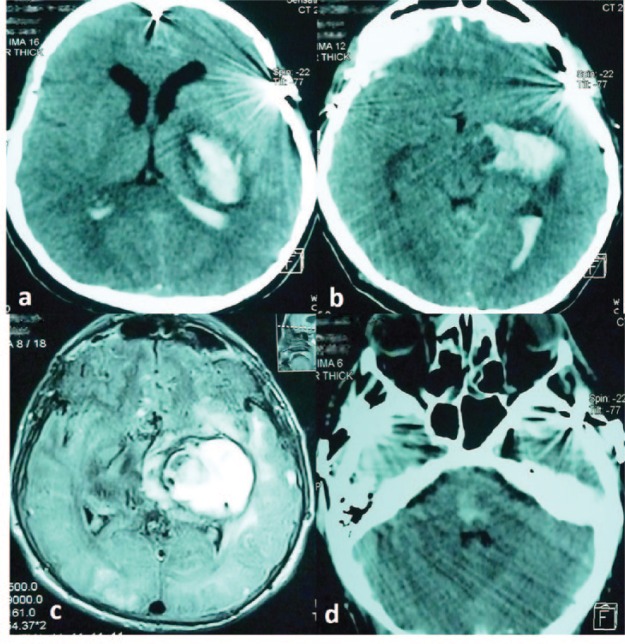
MRI and computed axial tomography (CT) of brain. (a, b, and c) Hemorrhage in the left putamen and internal capsule and intraventricular hemorrhage. (d) Hemorrhage at the medial pontine level.

Five years later after initial presentation (2013), the patient presented with a sudden loss of consciousness with right hemiparesis, so intensive care management was required. Neuroimaging showed a wide left putaminal hemorrhage extending to the subcortical white matter in addition to bleeding in the brain stem, with invasive bleeding of the ventricular system. Eventually, the patient developed generalized tonic-clonic seizures. The patient’s level of consciousness deteriorated to coma and he later reached a persistent vegetative state (Figure 5).

## Discussion

In DAVFs, the manner of venous drainage is what determines the severity of symptoms and allows classification between benign and aggressive fistulas. Aggressive fistulas are those fistulas that have a pattern of retrograde flow with drainage to venous sinus and cortical veins, and the fistulas with CVD (direct fistulas) are included in this group. These aggressive fistulas are classified as types II and III in the Borden classification and types IIb, IIa+b, III, IV, and V in the Cognard classification, and this is precisely the group that has a very aggressive clinical course,^15,26^ as was seen in our patient whose fistula eventually became type V.

The initial symptoms in DAVFs are due to the dilation of superficial facial blood vessels and consist mainly of headache and tinnitus. In other words, they meet the criteria of a secondary headache.^27^ Subsequently, blurred vision and bilateral papilledema occur, which correspond to clinical manifestations of intracranial hypertension, being in this case the underlying mechanism an alteration of venous drainage. This impaired venous drainage could be induced by venous hypertension as a result of dural fistulas.^28^ In the Cognard classification, type II fistulas with venous drainage induced intracranial hypertension in 20% of cases.^12^ A study by the same author included 13 patients with DAVF, 9 of which had intracranial hypertension as the first clinical manifestation (7 only with intracranial hypertension and 2 accompanied by tinnitus). The remaining 4 had other symptoms as the first clinical manifestations (2 with tinnitus, 1 with dilated superficial facial veins, and 1 with seizures). However, this group of patients ended up developing intracranial hypertension later on. The appearance of these symptoms was from 6 months to 13 years.^28^ Another factor taken into account was the type of venous drainage based on the revised classification of Djinjian and Merland, which found that, of the 13 patients, 11 corresponded to type II (either type IIa, IIb, or IIa+b).^28^ Similarly, our case was initially classified as type IIa by the Cognard classification.

Following the evolution in our patient, the MRI showed diffuse white matter changes. Kwon et al studied 27 patients with DAVF, 15% of which showed white matter hyperintensity. These changes on the MRI were accompanied by dilated cortical vessels and leptomeningeal enhancement, so these findings are prognostic factors; therefore, it is necessary to have an urgent therapy when DAVF has leptomeningeal enhancement,^29^ such as was observed in the MRI in our patient.

The pyramidal symptoms in our patient were the result of a compressive cervical myelopathy secondary to a spinal venous drainage of the dural fistula. In the series of case studies by Brunereau et al, 12 patients with DAVF and spinal venous drainage were investigated. They were classified into 2 groups, the first one consisting of 6 patients with progressive ascending myelopathy. The average age in the group with myelopathy was 55 years. All of these patients had sensory-motor deficits in the lower extremities along with urinary dysfunction. Three patients demonstrated the presence of a central cervical hyperintensity signal (finding that was very similar to that found in our patient).^3^ The spinal angiography showed no abnormalities during the arterial phase. However, venous drainage of the artery of Adamkiewicks was not seen in 3 cases. The cerebral angiography demonstrated a DAVF with drainage into the spinal venous sinus in 1 case, into the left superior petrosal sinus in 3 cases, and into the tentorium of the cerebellum in 2 cases. DAVFs were fed by branches of the internal and external carotid arteries. The initial venous drainage reached the perimesencephalic, peripontine, or peribulbar veins and descended into the cervical spinal perimedullary veins. In 1 case, it reached the anterior spinal vein, and in 5 cases, it reached the anterior and posterior spinal veins, but they did not drain into the venous plexus cervical epidural, and had a slower and more extensive venous drainage, reaching the thoracic region, including veins of the cauda equine. This resembles what was found in our patient, whose drainage was toward the perimedullary venous plexus and the anterior spinal vein. However, our patient had no drainage to lower levels. Despite this fact, the patient developed myelopathy, which makes our case different from the series of cases of Brunereau et al.^3^ It is proposed that the pathophysiological mechanism for the development of myelopathy is venous hypertension in the spinal cord.^3,30^ According to the Cognard classification, 50% of type V DAVFs cause progressive myelopathy.^12^

Regarding the evolution of DAVF in our patient, we saw a conversion of a benign fistula into an aggressive one. Kim et al observed the natural evolution of fistulas in 112 patients. This group consisted of patients who had refused to be treated and those who had established treatment failures. Ninety-nine patients had benign fistulas (Borden type I), 13 patients had aggressive fistulas (Borden type II/III); of these, 34 were studied with angiography (32 with Borden type I and 2 with Borden type II/III), showing a conversion of benign fistulas into aggressive ones in 4.0% of cases. The time for conversion to occur was 5 to 23 months with a mean of 12.3 months.^31^ This is similar to what happened with our patient, where there was the conversion of a benign fistula into an aggressive one after about 12 months. This conversion was associated with occlusion of the ipsilateral vein drainage.^31^ This finding was also observed by Satomi et al, who reported a progression of benign fistulas into aggressive ones in 2 of 117 patients. As in the previous work, conversion was associated with the presence of CVD due to venous thrombosis.^22^ With the case studies described above, we can see that the conversion of benign into aggressive DAVF is a rare phenomenon. A systematic review showed that 2% of benign DAVFs have a risk of developing CVD and thereby converting into an aggressive fistula.^12,22^

The persistence of CVD has been studied by van Dijk et al,^15^ in a cohort study of 118 patients with DAVFs, for which treatment was instituted in 101 cases. Three patients were lost to follow-up. Of the remaining 14 untreated patients and 6 partially treated, 7 suffered an intracranial hemorrhage and 9 patients died. The median follow-up in this group was 4.3 years. The annual death rate due to the persistence of CVD in DAVF was 10.4%, and the risk of a hemorrhagic or nonhemorrhagic neurological deficit was 8.1% and 6.9%, respectively. The case of our patient is part of the persistence group of CVD with hemorrhagic stroke.

There are several factors that come together in the development of dementia in patients with DAVF, the principal factor being CVD, which produces venous congestion and hypertension, leading to congestive encephalopathy. This venous hypertension reduces the normal gradient pressure between arteries and veins, which occurs most often in the deep venous system. Because of this, the white matter is the most vulnerable to venous congestion, leading to venous ischemia. This involvement of the white matter leads to the development of subcortical dementia^24,31-33^ and hemorrhagic stroke. The evaluation of venous hypertension is essential because it allows for the appropriate planning for treatment of dural fistulas.^34^

Dementia resulting from venous hypertensive encephalopathy was studied in the series of Hurst et al, where 40 patients with DAVF were studied, of which 12.5% had progressive deterioration of cognitive functions as early as 3 months up to more than a year. In all cases, MRI showed abnormalities in parenchymal cerebral regions, which were remote from the site of the DAVF with areas of edema or venous ischemia. In 4 patients, the fistula drained to the transverse venous and sigmoid sinuses. In all the cases, there was reflux of the adjacent dural sinus and cortical veins. Thus, the deterioration of the drainage of the deep and superficial venous system became a risk factor for the conversion to aggressive fistulas.^33^ Dementia caused by dural fistula may present with rapidly progressive dementia.^16,17,21,32,35^

The natural evolution of the DAVF depends mainly on 2 factors, one is the manner of the venous drainage, particularly with CVD, and the second is the presence or absence of aggressive clinical presentation symptoms. A recent study showed that patients who had a hemorrhagic stroke have suffered a mortality up to 7 years after admission.^22-24^ Söderman et al showed that the annual risk of bleeding was 7.4% in patients presenting with intracranial bleeding when compared with those who had no bleeding (1.5%).^25,36^ Cognard et al reported a dramatic increase in the incidence of aggressive clinical presentation in those with high-grade DAVF, when compared to those who had low-grade DAVF (89% vs 10%, respectively).^12^ This would explain why, in our case, the patient initially had few symptoms. However, later clinical events became more serious, resulting in a hemorrhagic stroke.

The final evolutionary stage of our patient was a persistent vegetative state. In the study by Kuwayama et al of 145 intracranial fistulas, 38 were aggressive lesions presenting with bleeding, seizures, and symptoms of intracranial hypertension. After treatment, a complete obliteration in 66% of cases and a partial obliteration in 34% was achieved. Eight percent progressed to a vegetative state, and 3% died due to acute myocardial infarction.^37^ As we can see, progression to a vegetative state is rare. As happened in our case, failing to achieve a complete obliteration of the aggressive fistula, the outcome was a persistent vegetative state.

**Figure 6.**
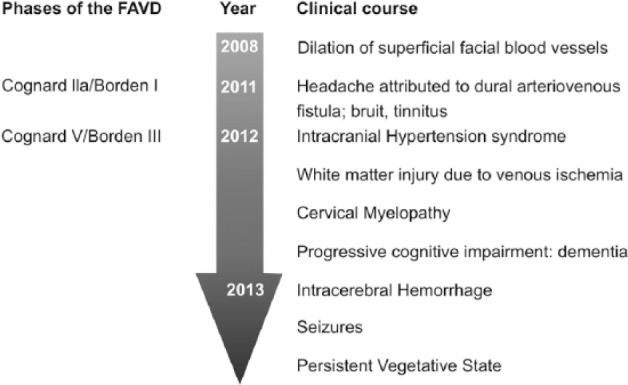
Evolutionary history of dural fistula. This patient had different clinical manifestations both in their presentation and in their evolution.

In our patient, the cause of multiple fistula could not be identified. The clinical variability in addition to the DAVF conversion from low to high makes it a rare and unique case, since as we mentioned, in the literature there are no clinical symptoms reported with symptoms as abundant as those that presented in our patient’s case (Figure 6). Dural fistulas may present with different clinical neurology both in their presentation and in their evolution (Table 3). They may mimic other diseases and sometimes result in problematic differences in diagnosis. The objective beyond determining the cause or mechanism of production is to detect early and to make a correct diagnosis by offering adequate treatment. However, one must consider the possibility of recurrences despite embolization and the possibility of fistula conversions from low to high. Though generally rare, this pathology becomes a real challenge for neurologists.

**Table 3.** Baseline Demographics, Clinical Characteristics, and Image Assessments of DAVF.

Reference	Number of Patients/Analyzed Patients	Demographics/Follow-up (years) and Main Objective of Study	Initial Clinical Presentation and Signs or Symptoms (%)	Classification: Borden or Cognard (%)	CVD (%)	MR Images (%)	Outcome (%)
Söderman et al^36^	163/85	Males 58%	*Intracranial hemorrhage* (37.6):	Borden II (37.6)	CVD 100	Not reported	Second hemorrhage (9.3)
		Mean age at diagnosis 58	1. Subarachnoid (15.3)	Borden III (62.4)			First hemorrhage (1.8)
		Mean follow-up 25	2. Cerebral or cerebellar (20)				Death (1)
		Natural course of untreated DAVFs with CVD	3. Subarachnoid and intracerebral (1.2)				
			4. Intraventricular bleed (1.2)				
			*Non–intracranial hemorrhage* (62.3):				
			1. Bruit (25.9)				
			2. Asymptomatic (14.1)				
			3. Another symptoms (21.1)				
			4. Progressive dementia (1.2)				
van Rooij et al^38^	91/29	Males 24	1. Intraparenchymal or subarachnoid hemorrhage (62)	Borden III (100)	1. CVD (69)	1. Dilated peripheral cortical veins (13.8)	1. Obliteration spontaneously (7)
		Mean age 53.9	2. Seizures (14)		2. Cortical cerebellar veins (17.2)	2. Occlusion of the transverse/sigmoid sinus (13.8)	2. Complete occlusion with embolization alone (48.1)
		Mean follow-up 12	3. Visual symptoms (7)		3. Perimesencephalic or peripontine veins (13.8)		3. Complete occlusion with embolization + surgery (24.2)
			4. Pulsatile bruit (3)				4. Complete occlusion with surgery (17.2)
			5. Asymptomatic (14)				5. Unknown (3.5)
van Dijk et al^15^	118/20	Males 11	1. Intracranial hemorrhage (25)	Borden II (75)	CVD (100)	Not reported	1. Severely disabled (5)
		Mean age 56.7 (one child 3 years old)	2. NHND (45)	Borden III (25)			2. Remained stable with moderate disabilities (25)
		Mean follow-up 4.3	3. Generalized seizures (10)				3. Resolution of the preexisting symptoms (25)
		Persistence of CVD	4. Cranial bruit (10)				4. Death (45)
			5. Orbital phenomena (5)				5. Second hemorrhage (15)
			6. Asymptomatic (5)				6. Progressive dementia syndrome (20)
							7. NHND (10)
Brunereau et al^3^	258/12	Males 7	*Myelopathy* (50)	Cognard V (100)	1. Spinal venous drainage + venous drainage (41.7)	*Myelopathy*	Not reported
		Mean age 52.5	1. Progressive sensorimotor deficit with urinary dysfunction		2. Spinal venous drainage (58.3)	1. T1-weighted MR images of the spinal cord perimedullary flow voids (25)	
		Retrospective	*Without myelopathy* (50)			2. T2-weighted images perimedullary flow voids and a central hyperintense signal of the cervical spinal cord (8.3)	
		DAVF with spinal venous drainage	1. Subarachnoid hemorrhage + meningeal syndrome (25)				
			2. Subarachnoid hemorrhage + coma (16.7)				
			3. Transient aphasia (8.3)				
Hurst et al^33^	40/5	Males 5	1. Memory loss, encephalopathy + chronic headaches + bruit (20)	Cognard IIa+b (60)	1. Retrograde into adjacent dural sinuses + CVD (60)	Enlarged vessels over hemispheric	Partial occlusion with one embolization: clinical improvement in mental status (40)
		Mean age 66	2. Dementia + chronic headaches + bruit (80)	Cognard III (20)	2. Retrograde into cortical veins (20)	Surface + increased T2-weighted signal (60)	Partial occlusion with second embolization: remission of cognitive symptoms (60)
		Follow-up >1		Cognard IV (20)	3. Retrograde into cortical veins + venous dilatation (20)	MR angiogram: high flow veins (40)	
		Diffuse encephalopathy or a chronic dementing process					
Cognard et al^28^	120/13	Males 7	Isolated symptoms of intracranial hypertension (53.8)	Cognard I (15.4)	Dural venous sinus with antegrade flow (15.4)	Chronic tonsillar herniation + syringomyelia (7.7)	Chronic tonsillar herniation asymptomatic (15.4)
		Mean age 50	Associated intracranial hypertension with tinnitus (15.4)	Cognard IIa (38.5)	Dural venous sinus with retrograde flow (38.5)	Chronic tonsillar herniation (7.7)	Acute tonsilar herniation (coma) after lumboperitoneal shunting (7.7)
		Mean follow-up 7.5	Tinnitus (15.4)	Cognard IIb (7.7)	Dural venous sinus with antegrade flow and CVD (7.7)	Not reported (84.6)	Acute tonsilar herniation (death) after lumboperitoneal shunting (7.7)
		Isolated or associated signs of intracranial hypertension	Seizure (7.7)	Cognard IIa+b (30.7)	Dural venous sinus with retrograde flow and CVD (30.7)		Acute confusion after lumbar puncture regressive after embolization (7.7)
			Frontal superficial veins dilatation (7.7)	Cognard IIa, IIa, IIb (DAVF multiple) (7.7)	DAVF multiple (7.7)		Not reported (61.5)
Kwon et al^29^	27/27	Male 12	Ocular symptoms + hemorrhage (3.7)	Borden I (29.7)	Dural venous sinus (29.7)	Flow void clusters (82)	Not reported
		Mean age 52	Ocular symptoms + tinnitus (7.4)	Borden II (44.4)	CVD (70.3)	Engorged ophthalmic vein (30)	
		Retrospective	Ocular symptoms (29.7)	Borden III (22.2)		White matter hyperintensity (15)	
		Identify MR imaging finding differences between DAVF types classified on the basis of venous drainage patterns	Hemorrhage (14.8)	DAVF multiple (3.7)		Intracranial hemorrhage (30)	
			Tinnitus (11.1)			Dilated leptomeningeal or medullary vessels (44)	
			Seizure (7.4)			Venous pouch (7)	
			Focal neurologic deficit + hemorrhage (7.4)			Leptomeningeal or medullary vascular enhancements (41)	
			Focal neurologic deficit (3.7)			MR angiograpic fistula and venous flow-related enhancement (91)	
			Altered mentality + hemorrhage (3.7)			Prominent extracranial vessels (36)	
			Altered mentality (3.7)				
			Intracranial hypertension (3.7)				
			Asymptomatic (3.7)				

Abbreviations: CVD, cortical venous drainage; DAVF, dural arteriovenous fistulas; MR, magnetic resonance; NHND, nonhemorrhagic neurologic deficit.
